# Consensus clinical management guidelines for Niemann-Pick disease type C

**DOI:** 10.1186/s13023-018-0785-7

**Published:** 2018-04-06

**Authors:** Tarekegn Geberhiwot, Alessandro Moro, Andrea Dardis, Uma Ramaswami, Sandra Sirrs, Mercedes Pineda Marfa, Marie T. Vanier, Mark Walterfang, Shaun Bolton, Charlotte Dawson, Bénédicte Héron, Miriam Stampfer, Jackie Imrie, Christian Hendriksz, Paul Gissen, Ellen Crushell, Maria J. Coll, Yann Nadjar, Hans Klünemann, Eugen Mengel, Martin Hrebicek, Simon A. Jones, Daniel Ory, Bruno Bembi, Marc Patterson, on behalf of the International Niemann-Pick Disease Registry (INPDR)

**Affiliations:** 1 Institute of Metabolism and System Research, University of Birmingham, Birmingham, UK; 2 AMC Hospital of Udine, Udine, Italy; 3 Royal Free London NHS Foundation Trust, London, UK; 4 Vancouver General Hospital, Vancouver, Canada; 5 Hospital Sant Joan de Deu, Barcelona, Spain; 6 INSERM U820, Université de Lyon, Faculté de Médecine Lyon-Est, Lyon, 69372 France; 7 Royal Melbourne Hospital, Parkville, Australia; 8 University Hospitals Birmingham NHS Foundation Trust, Birmingham, UK; 9 Department of Pediatric Neurology, Reference Center of Lysosomal Diseases, Trousseau Hospital, APHP, GRC ConCer-LD, Sorbonne Universities, UPMC University 06, Paris, France; 10 Universitatsklinikum Tubingen Institut fur Medizinische Genetik undangewandte Genomik, Tubingen, Germany; 11 Niemann-Pick UK, Washington, UK; 12 Salford Royal NHS Foundation Trust, Salford, UK; 13 MRC Laboratory for Molecular Cell Biology, London, UK; 14 Children’s University Hospital, Dublin, Republic of Ireland; 15 Hospital Clinic de Barcelona, Barcelona, Spain; 16 Hopital Universitaire Pitie Salpetriere, Paris, France; 17 Universitatsklinikum Regensburg Klinik und Poliklinik fur Chirurgie, Regensburg, Germany; 18 Universitatmedizin Mainz, Mainz, Germany; 19 Charlies University in Prague, Prague, Czech Republic; 20 Central Manchester University Hospitals NHS Foundation Trust, Manchester, UK; 21 University of Washington School of Medicine, Seattle, USA; 22 Mayo 1290 Clinic Department of Pediatric and Adolescent Medicine, Minnesota, USA

**Keywords:** Niemann-Pick Type C, NPC, Guidelines, Diagnosis, Management

## Abstract

Niemann-Pick Type C (NPC) is a progressive and life limiting autosomal recessive disorder caused by mutations in either the *NPC1* or *NPC2* gene. Mutations in these genes are associated with abnormal endosomal-lysosomal trafficking, resulting in the accumulation of multiple tissue specific lipids in the lysosomes. The clinical spectrum of NPC disease ranges from a neonatal rapidly progressive fatal disorder to an adult-onset chronic neurodegenerative disease. The age of onset of the first (beyond 3 months of life) neurological symptom may predict the severity of the disease and determines life expectancy.

NPC has an estimated incidence of ~ 1: 100,000 and the rarity of the disease translate into misdiagnosis, delayed diagnosis and barriers to good care. For these reasons, we have developed clinical guidelines that define standard of care for NPC patients, foster shared care arrangements between expert centres and family physicians, and empower patients. The information contained in these guidelines was obtained through a systematic review of the literature and the experiences of the authors in their care of patients with NPC. We adopted the Appraisal of Guidelines for Research & Evaluation (AGREE II) system as method of choice for the guideline development process. We made a series of conclusive statements and scored them according to level of evidence, strengths of recommendations and expert opinions. These guidelines can inform care providers, care funders, patients and their carers of best practice of care for patients with NPC. In addition, these guidelines have identified gaps in the knowledge that must be filled by future research. It is anticipated that the implementation of these guidelines will lead to a step change in the quality of care for patients with NPC irrespective of their geographical location.

## Background

Niemann-Pick Type C (NPC) disease is a rare genetic disease whose clinical spectrum ranges from a fatal antenatal disorder to an adult-onset chronic neurodegenerative disease. The rarity of the disease and the scarcity of expertise translate into misdiagnosis, delayed diagnosis and barriers to adequate care. This results in additional physical, psychological and intellectual impairments, inadequate or inappropriate treatment, loss of confidence in the healthcare system, and patient disempowerment, even though the diagnosis of NPC is compatible with improved quality of life if a diagnosis is made promptly and appropriate comprehensive management is instituted. There is as yet no disease-specific curative therapy available, and the disease usually progresses to premature death. The mainstay of management is symptomatic supportive therapy using multidisciplinary and multiprofessional teams of experts. Some countries have national standard operating procedures to improve the care of NPC patients, but the NPC community, represented by the International Niemann-Pick disease Alliance (INPDA), has not previously initiated the development of a comprehensive disease management guidelines to provide a resource for the multidisciplinary team, and to support patients and their primary professional caregivers on the current diagnosis, treatment, monitoring and outcome measures for patients with NPC. This document represents general guidelines, which in the opinion of the authors can inform care providers about the needs of patients with NPC in order to provide equitable and improved care, define standard of care for NPC patients, foster shared care arrangements between expert centres and family physicians, and empower patients. The guidelines refer to the management of patients suspected or diagnosed with NPC disease at any age. These guidelines should be of value to: a) specialist centres, other hospital-based medical teams and other staff involved with the care of NPC disease patients, b) family physicians and other primary caregivers and c) patients and their families. The guidelines have been developed by experts with extensive experience of European, Australian and North American healthcare systems and populations. However, they might equally be applicable to any country that operates similar healthcare services. It is anticipated that implementation of these guidelines will lead to a step change in the quality of care for patients with NPC.

## Methods

These guidelines have been developed by expert physicians, geneticists, allied healthcare professionals and patient support groups involved in the International Niemann-Pick Disease Registry (INPDR) project (www.inpdr.org),which is supported by the EU Directorate General for Health and Consumers (DG-SANCO) via the Consumers, Health, Agriculture and Food Executive Agency (CHAFEA). The INPDR consortium comprises 27 partners from 13 countries in Europe, Australia, Canada and the United States of America. One of the goals of the INPDR is to support equitable care of Niemann-Pick disease patients by standardizing the quality of care all patients receive. In addition, the European Metabolic Reference network (MetabERN) has adopted this guideline for the management of NPC patients within the network.

The Guidelines Development Group (GDG) consisted of expert representatives from a range of professional groups including paediatric and adult neurologists, paediatric and adult metabolic specialists, psychiatrists, epidemiologists, clinical biochemists, geneticists, specialist metabolic dieticians, physiotherapists, psychologists, specialist nurses and patient support group representatives. The GDG Committee agreed the remit of the guidelines and selected a list of guidelines topics for development.

A systematic literature review on NPC in the last 10 years until May 2017 was carried out using Medline, MedLink, Embase and the Cochrane Library. Relevant papers, which were previously published were considered by the GDG members as important were included. Searches were limited to English language publications only. The initial search identified 879 reference abstracts, of which 276 were accepted as relevant after the first screen. References related to a single topic (i.e., Epidemiology, Genetics, Pathophysiology, Clinical Diagnosis, Laboratory, Imaging, Therapy, Recommendations) were pulled together and the GDG was divided into subgroups aimed to critically appraise references devoted to a specific topic. The committee met twice (June 2016, Birmingham, UK and September 2016, Rome, Italy) and corresponded by email on a regular basis throughout the duration of the guideline development. During the first workshop, the GDG adopted the second version of the Appraisal of Guidelines for Research & Evaluation (AGREE II) system as methodological reference in order to meet the guideline development standards outlined in the AGREE II system: howewer, our guideline didn’t partially or completely met 5/23 items outlined in the AGREE II system, and we haven’t calculated quality scores for all appraisal items [1].

Relevant papers were evaluated by members of the GDG before the evidence was considered. Section leaders individually assessed the literature selected and wrote a short document describing the study findings and related recommendations. All GDG members discussed the draft documents. Evidence levels were classified in accordance with the Grading of Recommendations, Assessment, Development and Evaluations (GRADE) methodology and recommendations were graded from A to C (Table 1). In addition, for the adoption of recommendations, we structured a panel of experts that represented group of specialists caring for NPC patients and used the Delphi method for the development of our guidelines. In total, 25 persons participated, and after the first round of Delphi consensus two statements required substantial revision and the expert opinion expressed in the guidelines were based on the revised statements.

**Table 1:** Evidence levels and strength of recommendations

Item	Definition
Level of evidence	
A. High-quality evidence	Further research is unlikely to change our confidence in the estimate of effect. Consistent evidence from the RCTs without important limitations or exceptionally strong evidence from observational studies.
B. Moderate-quality evidence	Further research is likely to have an important impact on our confidence in the estimate of effect and may change the estimate. Evidence from RCTs with important limitations (inconsistent results, methodologic flaws, indirect or imprecise), or very strong evidence from observational studies.
C. Low-quality evidence	Further research is very likely to have an important impact on our confidence in the estimate of effect and is likely to change the estimate. Evidence for at least one critical outcome from observational studies, case series, or from RCTs with serious flaws, or indirect evidence, or expert’s consensus.
Strength of recommendation	
1. Strong recommendation	Recommendation can apply to most patients in most circumstances.
2. Weak recommendation	The best course of action may differ depending on circumstances or patient or society values. Other alternatives may be equally reasonable.

The guidelines will be published in an open access journal and made freely accessible through the INPDR and INPDA website. These guidelines will be revised every 3–5 years to reflect new data pertaining to future research findings, new therapies and the development of diagnostic methods. The development of these guidelines was made without external financial support from industries involved in the manufacturing of therapies for NPC disease. Competing interests of members of the guideline development group have been recorded in writing and addressed. Developing treatment guidelines in an objective and scientific manner for a rare disease is challenging owing to the lack of randomized controlled trials (RCT). We have attempted to apply all the AGREE II domains in our guidelines development. However, as the methodology was developed for common disorders, where there is wealth of evidence in a form of RCT, a large number of patients cared by large number of clinical units, despite our best effort, we found it difficult to apply AGREE II in full for an ultra-rare disorder. The small number of patients available for clinical studies, variability of the phenotype and constraints on funding limit the opportunities to mount clinical trials. We have therefore created guidelines using the best available data, however imperfect it may be.

## Defintion and epidemiology

### Definition of NPC

***Statement #1:*** *NPC is a progressive and life limiting autosomal recessive disorder caused by mutations in either the NPC1 or NPC2 gene. Mutations in these genes are associated with abnormal endosomal-lysosomal trafficking, resulting in the accumulation of multiple lipids in the lysosomes. Disease onset occurs through the lifespan, from antenatal life to maturity.*

- *Strength of recommendation: 1*
- *Level of evidence: B*
- *Experts opinion: completely agree (94%), mostly agree (6%), partially agree (0%), mostly disagree (0%) and completely disagree (0%).*

Niemann-Pick disease type C (OMIM#257220; OMIM#607625) is a lysosomal storage disorder caused by mutations in either *NPC1* or *NPC2* genes [2–4]. The two genes code for their respective proteins, NPC1 and NPC2 [5, 6]. The two proteins, working in a coordinated manner, are believed to be involved in the cellular trafficking of cholesterol [7] and other lipids in the late endosomal/post-lysosomal stage of lipid transport. Disease causing mutations in either gene result in tissue accumulation of multiple lipids (see Ref [8] for review). Both types of NPC are inherited in an autosomal recessive manner. NPC disease is a progressive disorder characterized by neuro-visceral manifestations that can present at any age from the perinatal period to maturity. Life expectancy in patients with NPC disease varies with the age of onset of the disease and ranges from a few days to several decades [9–13].

### How common is NPC disease?

***Statement #2:*** *NPC disease is rare, with an estimated incidence of 1 case per 100,000 live births. The disease is pan-ethnic, with at least 95% of all disease due to mutations in the NPC1 gene and the remainder in the NPC2 gene.*

- *Strength of recommendation: 2*
- *Level of evidence: C*
- *Experts opinion: completely agree (65%), mostly agree (29%), partially agree (6%), mostly disagree (0%) and completely disagree (0%).*

Retrospective national expert centre based studies from Australia, the Netherlands, the UK, Portugal, Czech Republic, France and United Arab Emirates have reported an annual incidence varying between 0.25 and 2.20 per 100,000 live births [11, 14–18]. Published incidence data that includes information from prior to 1990 may underestimate the disease prevalence. These figures should be compared with those recently compiled from parallel large exome sequencing datasets [19]. In the latter study, when taking into account pathogenic mutations, the incidence rate at conception of classical clinical forms of NP-C was calculated to 1.12 per 100,000 (1/89,229). In addition, owing to better awareness and improved diagnostic methods, a significantly higher proportion of adult-onset cases have been diagnosed during the past 5 years [20], therefore a recalculation would likely approach a figure slightly higher than 1/100,000. Interestingly, in the Wassif et al. study, the inclusion of two variants of controversial pathogenicity would suggest a much higher incidence in the range of 1/40,000 of still unrecognized late onset, milder form [19]. Indeed, attenuated phenotypes may not be suspected clinically, or may be missed by the diagnostic laboratories.

## Clinical presentation

### How best can NPC disease be classified?

***Statement #3:*** *The clinical manifestations and life expectancy of NPC patients vary markedly with age of onset of the disease. In neonates and children, NPC may initially present as a systemic disease with subtle neurological manifestations, but for practical purposes, NPC is best classified according to the age of onset of neurological manifestations as follow:*

1. *visceral-neurodegenerative form*
*Early-infantile (< 2 years)*
2. *neurodegenerative form*
*Late-infantile (2–6 years)*
*Juvenile (6–15 years)*
3. *Psychiatric-neurodegenerative form*
*Adult (> 15 years)*

- *Strength of recommendation: 1*
- *Level of evidence: B*
- *Experts opinion: completely agree (50%), mostly agree (38%), partially agree (13%), mostly disagree (0%) and completely disagree (0%).*

The clinical spectrum of NPC disease ranges from a neonatal rapidly progressive fatal disorder to an adult-onset chronic neurodegenerative disease. Based on data gathered from a large cohort of French NPC patients, the age of onset of neurological symptoms predicts the severity of the disease and determines life expectancy [11]. Disease classifications based on the age of onset of the first (beyond 3 months of life) neurological symptom may be used to guide clinicians in providing day to day care, genetic counselling and estimate the trajectory of the disease course. There is an overlap between the neurological forms, as NPC disease comprises a continuum [20]. Relative distributions of the five age categories based on the national/international registry are listed in Table 2. In addition, atypical presentations such as: a) the fatal systemic perinatal form (foetal hydrops or early liver, multi-organ or respiratory failure) and b) initial systemic disease only (in infants and children with a variable latency before onset of neurological manifestations) constitutes a small, but significant, proportion of cases. The global contribution of these forms has rarely been calculated, and such patients are typically not enrolled in registries. Two features emerge from the compiled data presented in Table 2: the early infantile neurological onset form appears more frequent in Southern Europe, and 2) patients with an adolescent/adult onset neurological form seem to represent at least 20% of the cases of NPC and, owing to their longer survival, probably constitute the largest patient group in terms of disease prevalence [20].

**Table 2:** Distribution of clinical forms of NP-C disease in large cohorts

	Neonatal systemic fatal (%)	Early infantile neurological onset (%)	Late infantile neurological onset (%)	Juvenile neurological onset (%)	Adolescent/adult neurological onset (%)	Total no.
Early studies						
France+ European countries [70]	12	30	23	30	5	125
Spain [71–73]	7	37	21	25	11	57
Italy [74, 75]	7	26	32	23	12	43
France [11]	9	26	22	26	16	107
Recent studies						
Germany [22]	3	3	35	54	5	37
Czech Republic [12]	6	17	24	37	17	54
UK [13]	5	6	39	32	19	132
European countries [20]	–	11	31	31	27	145

### Is the clinical presentation different in specific age groups?

#### Pre/perinatal (< 2 months)

***Statement # 4:*** *NPC manifests in the pre/perinatal age group primarily as liver disease presenting with prolonged cholestatic jaundice, hepatosplenomegaly and in some cases acute liver failure, with or without pulmonary disease.*

- *Strength of recommendation: 1*
- *Level of evidence: B*
- *Experts opinion: completely agree (60%), mostly agree (40%), partially agree (0%), mostly disagree (0%) and completely disagree (0%).*

NPC disease presentation during the neonatal period varies from subject to subject, with the commonest presentation being prolonged jaundice and mild hepatosplenomegaly. In the majority of cases, jaundice resolves spontaneously by 3–4 months of age, while organomegaly persists to a variable degree. Neurological symptoms develop later, with a delay varying between a couple of months and the childhood period, or even later in a few cases. However, in about 8–9% of the cases, the hepatic manifestations may progress rapidly to acute liver and/or multi-organ failure and subsequently lead to death within 6 months. In some circumstances, the initial presentation may be foetal ascites/hydrops. The rapidly progressing cohort may have associated neurological presentations such as failure to thrive and hypotonia [10].

#### Early infantile (2 months to < 2 years):

***Statement #5:*** *Hypotonia and delay in developmental motor milestones characterize the neurological manifestation of NPC in early infancy. Hepatosplenomegaly and/or a neonatal prolonged jaundice are almost invariably noted.*

- *Strength of recommendation: 1*
- *Level of evidence: B*
- *Experts opinion: completely agree (53%), mostly agree (40%), partially agree (7%), mostly disagree (0%) and completely disagree (0%).*

*Hypotonia may be due to cerebral and/or peripheral nerve pathology in these early infantile forms. In the latter case, distal motor limb deficit may be clinically obvious. Communication is initially well preserved. Vertical supranuclear gaze palsy (VSGP) may be present, but is difficult to recognize.*

#### Late infantile (2 to < 6 years)

***Statement #6:*** *Clumsiness, gait disturbance and fine motor skill impairments characterize this age of onset of the disease. Speech delay, a history of neonatal cholestasis and variable visceromegaly may be noted. VSGP is typically present, but is often unrecognized. The first symptoms may be gelastic cataplexy (sometimes associated with narcolepsy) or sensory deafness. Epilepsy is quite frequent in subsequent evolution in this group.*

- *Strength of recommendation: 1*
- *Level of evidence: B*
- *Experts opinion: completely agree (67%), mostly agree (33%), partially agree (0%), mostly disagree (0%) and completely disagree (0%).*

#### Juvenile (6 to 15 years)

***Statement #7:*** *Juvenile-onset is the 2nd most frequent presentation of NPC and manifests as cognitive impairment (lagging behind peers in school, language and learning difficulties), coordination problems (clumsiness, frequent falls, progressive ataxia and dystonia) and VSGP.*

- *Strength of recommendation: 1*
- *Level of evidence: B*
- *Experts opinion: completely agree (60%), mostly agree (40%), partially agree (0%), mostly disagree (0%) and completely disagree (0%).*

#### Adult (> 15 years)

***Statement #8:*** *Adolescent and adult-onset NPC patients may represent up to a third of all NPC patients. Cognitive impairment invariably occurs and tends to present with higher rates of psychiatric illness co-existing with neurological manifestations. Diagnostic delay is common, but is minimised if the characteristic VSGP is identified.*

- *Strength of recommendation: 1*
- *Level of evidence: B*
- *Experts opinion: completely agree (27%), mostly agree (67%), partially agree (7%), mostly disagree (0%) and completely disagree (0%).*

The age of onset of NPC varies significantly across the lifespan (Table 3) [11], although increasingly patients are recognized as presenting with late-onset illness in adolescence, early and mid-adulthood and may present as late as the 7th decade [21]. Early development is often completely normal achieving all developmental milestones appropriate to their age. In a large international prospective registry, the adolescent and adult-onset form occurred in 27% of all NPC patients [20]. Patients in this age group are less likely to present with seizures, gelastic cataplexy and diagnosed visceral disease. Typical presentation is a history of progressive ataxia/dystonia, and/or cognitive decline, and/or atypical psychotic symptoms, often associated to vertical gaze palsy at clinical examination [11, 22–25]. Other reported symptoms dysarthria and dysphagia are also very frequent but occur later. Some patients may have previous symptoms that have begun several years before onset of the chronic neurodegenerative disease such as undiagnosed hepatomegaly or splenomegaly with spontaneous remission in childhood, learning disorder in childhood, and hearing defect. In late-onset patients, diagnostic delay is common and is often 5 years or more, although this delay may be minimised when the more specific symptom of VSGP is recognised [26]. Despite the diagnostic utility of VSGP, it may not be present if patients are examined early in the course of the disease, and its absence should not rule out the diagnosis of NPC.

**Table 3:** Summary of Clinical signs and symptoms in NP-C, by age of onset

Age at onset	Systemic manifestations	Neurological/psychiatric manifestations
Pre−/peri-natal (< 2 months)	Foetal ascites/hydrops	Hypotonia
	Hepatosplenomegaly	
	Cholestatic jaundice	
	Thrombocytopenia	
	Pulmonary disease	
	Liver failure	
	Failure to thrive	
Early-infantile (2 m to < 2 yrs.)	Hepatosplenomegaly or Splenomegaly (isolated or with neurological manifestations)	Central hypotonia Delayed developmental motor milestones, speech delay
	Prolonged neonatal jaundice	Dysphagia, spasticity
		VSGP
Late-infantile (2 to < 6 yrs.)	Hepatosplenomegaly or Splenomegaly (isolated or with neurological manifestations)	Developmental delay/regression, speech delay
	History of prolonged neonatal cholestatic jaundice	Clumsiness, Frequent falls,
		Progressive ataxia, dystonia, dysarthria, dysphagia,
		Seizures (partial/generalized)
		Cataplexy
		VSGP
		Hearing loss
Juvenile (6 to 15 yrs.)	Hepatosplenomegaly or Splenomegaly (isolated or with neurological manifestations; often not present)	Poor school performance, learning disability. Loss of language skill
		Frequent falls, clumsiness
		Progressive ataxia, dysarthria, dystonia, dysmetria, dyskinesia, dysphagia
		VSGP
		Gelastic cataplexy Seizures
		Behavioural problems
Adolescent/adult (> 15 yrs.)	Splenomegaly (often not present; isolated in very rare cases)	Cognitive decline, dementia, learning disability Psychiatric signs: Schizophrenia (psychosis), depression.
		Clumsiness, progressive motor symptoms, tremor, ataxia, dystonia/dyskinesia, dysarthria, dysphagia VSGP

Pre-senile cognitive impairment, prominently affecting memory and executive function, invariably occurs in adolescent and adult-onset NPC patients [23, 26–28]. Furthermore, up to one third of adolescent and adult patients may present with psychiatric symptoms such as psychosis (paranoid delusions, auditory and/or visual hallucination) and depression which predate neurological manifestations and exhibit atypical features, including treatment- resistance [12, 26, 29, 30]. Up to one third of patients presenting with psychiatric illness, most commonly psychosis, may show a poor response to treatment. The combination of psychiatric and neurological presentation should raise the clinical suspicion of a diagnosis of NPC.

The NPC-suspicion index assists in the diagnosis of adult patients with NPC, with strong indicators including cognitive and psychotic symptoms, and the combination of neurological with psychiatric signs is highly suggestive of NPC [26, 31]. NPC patients may have initially a poorly specific presentation, but with accumulation of typical disorders, probability of diagnosis strongly increases, as illustrated by the series of ataxia clinic patients with recessive disease and cognitive decline for whom NPC was genetically confirmed in one sixth of them [25].

### Conditions raising the suspicion of NPC and differential diagnosis

The symptoms and signs of NPC vary with age at disease onset. There are numerous conditions raising the suspicion of NPC and other age appropriate diseases should also be excluded.

***Statement #9:*** *In the first 2 years of life, history of prolonged neonatal jaundice, hepatosplenomegaly and/or developmental delay should raise the possibility of NPC. The differential diagnosis includes other causes of cholestatic jaundice, idiopathic neonatal hepatitis, Wolman disease, Niemann-Pick type A/B, Gaucher type III disease and Cerebrotendinous xanthomatosis with or without initial developmental delay.*

- *Strength of recommendation: 1*
- *Level of evidence: B*
- *Experts opinion: completely agree (50%), mostly agree (36%), partially agree (14%), mostly disagree (0%) and completely disagree (0%).*

***Statement #10:*** *From childhood to adolescence, neurological disease manifestations can be subtle, ranging from clumsiness and poor school performance to progressive ataxia, dysarthria and dystonia. Other age appropriate neurodegenerative disorders must be ruled out.*

- *Strength of recommendation: 1*
- *Level of evidence: B*
- *Experts opinion: completely agree (73%), mostly agree (27%), partially agree (0%), mostly disagree (0%) and completely disagree (0%)*

NPC can also manifest as isolated splenomegaly or hepatosplenomegaly and mimics NPB disease (same storage cells) or Gaucher disease. NPC should be considered in differential diagnosis in patients with unexplained isolated splenomegaly with or without hepatomegaly at any age.

***Statement #11*** *: Adult patients presenting with an atypical psychotic disorder or a progressive neurological syndrome including ataxia, dystonia, cognitive difficulties, dysarthria, or VSGP with or without splenomegaly should be tested for NPC. Other neurodegenerative disorders such as Huntington disease, Wilson disease, Cerebrotendinous xanthomatosis, GM1 or GM2 gangliosidoses and Friedreich Ataxia which mimic NPC must be ruled out. However, on the contrary to several of those diseases, in NPC patients, there is no peripheral neuropathy and brain MRI is normal or shows nonspecific abnormalities (mainly atrophy).*

- *Strength of recommendation: 1*
- *Level of evidence: B*
- *Experts opinion: completely agree (67%), mostly agree (33%), partially agree (0%), mostly disagree (0%) and completely disagree (0%).*

### NPC disease severity score

***Statements #12:*** *NPC-specific disease severity scores are useful adjuncts to clinical judgement in assessing disease burden, response to therapy and determining prognosis.*

- *Strength of recommendation: 1*
- *Level of evidence: B*
- *Experts opinion: completely agree (53%), mostly agree (40%), partially agree (7%), mostly disagree (0%) and completely disagree (0%).*

Clinical assessment of disease severity is dependent on the experience of the treating clinician, but such clinical judgement may not be reliable when the disease is rare and most clinicians have limited exposure to the condition. Three severity scoring systems and predictive models have been developed in an attempt to help the clinician accurately assess disease burden and monitor progression in time or stabilisation with therapy and estimate prognosis at an early stage [22, 24, 32]. These NPC–specific scales were based on neurological impairments that allow a calculation of a composite score to assess disease severity. Bearing in mind the resources available to most physicians in practice, we suggest the use of a modified version (Table 4) of the widely implemented and user-friendly model [24] though it has not been formally validated for treatment monitoring. No predictive model allows the unequivocal categorisation of patients into definite groups and predictive models are best viewed as useful adjuncts to clinical judgement. Regular reassessment of severity over the course of the disease is mandatory to assess the response to treatment.

**Table 4:** Clinical Severity assessment

I. Functional disability scale (Modified from Pineda et al. [24])	
Ambulation	Score
Normal	0
Clumsiness	1
Autonomous ataxic gait	2
Outdoor assisted ambulation	3
Indoor assisted ambulation	4
Wheelchair-bound	5
Manipulation	Score
Normal	0
Tremor	1
Slight dysmetria/dystonia (allows autonomous manipulation)	2
Mild dysmetria/dystonia (requires help for several tasks but is able to feed themselves)	3
Severe dysmetria/dystonia (requires assistance in all activities)	4
Language	Score
Normal	0
Delayed acquisition	1
Mild dysarthria (understandable)	2
Severe dysarthria (only comprehensible to some family members)	3
Non-verbal communication	4
Absence of communication	5
Swallowing	Score
Normal	0
Occasional dysphagia	1
Daily dysphagia	2
Nasogastric tube or gastric button feeding	3
Eye movements	Score
Normal	0
Slow ocular pursuit	1
Vertical ophthalmoplegia	2
Complete ophthalmoplegia	3
Seizure	Score
No	0
Yes, controlled by antiepileptic drugs	2
Yes, uncontrolled on two or more antiepileptic drugs of maximally tolerable dose	4
II. Neurocognitive Assessment	
Development (< 12 years old):	
O Normal	
O Mild learning delay	
O Moderate learning delay	
O Severe delay/plateau	
O Regression	
Memory (> 12 years old):	
O Normal	
O Mild impairment	
O Moderate	
O Difficult following commands	
O Unable to follow commands	

#### Is saccadic eye movement evaluation a measure of disease status?

***Statements #13:*** *Measures of horizontal saccadic function are robust objective measures of illness status and correlate with indices of brain structure.*

- Strength of recommendation: 2
- Level of evidence: B
- Experts opinion: completely agree (23%), mostly agree (23%), partially agree (46%), mostly disagree (8%) and completely disagree (0%).

Vertical saccadic gaze palsy is a clinical hallmark of the disease, whereas horizontal saccadic gaze deteriorates less rapidly and is a useful objective biomarker of disease severity illness. Horizontal saccadic gain, which correlates strongly with measures of pontine area and parietal eye field volume as measured on MRI, and self-paced saccades, an index of frontal eye field integrity, may be the most robust measure in adults [33, 34].

## Investigations

**Fig. 1:**
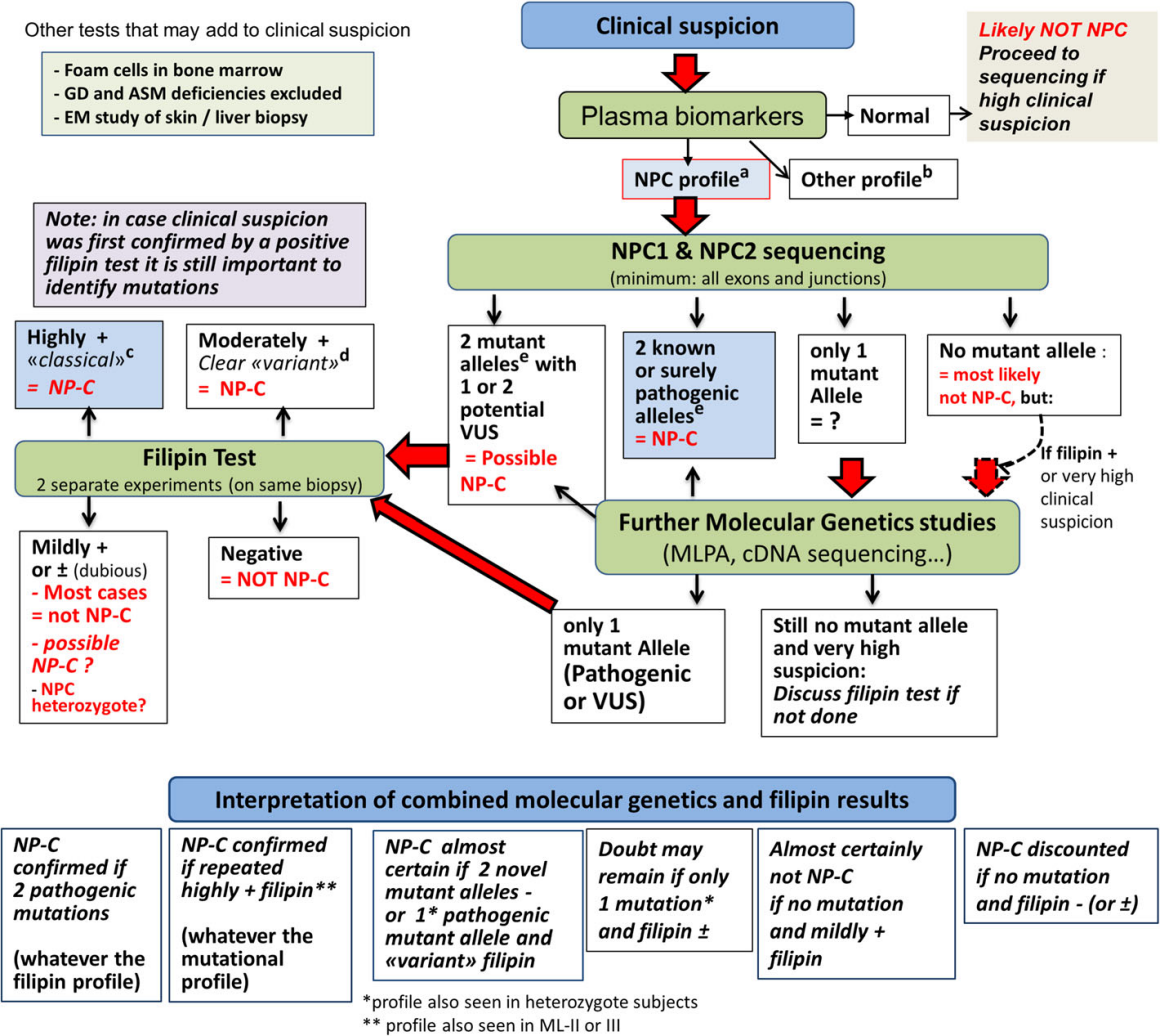
Niemann-Pick disease type C laboratory diagnosis algorithm. Modified from: Patterson et al. [36, 47]. Abbreviations: GD: Gaucher disease; ASMD: acid sphingomyelinase deficiency; EM: electron microscopy; VUS: variant of unknown significance; MLPA: Multiplex Ligation-dependent Probe Amplification (evaluates copy number changes, allows detection of large deletions or false homozygous status with a deletion on the other allele); lysoSM: lysosphingomyelin. ^a^Elevated cholestane-triol or bile acid derivative and/or lysoSM-509, with normal or slightly elevated lysoSM. ^b^Cholestane-triol also elevated in ASMD, acid lipase deficiency, cerebrotendinous xanthomatosis, certain neonatal cholestasis conditions. All lysoSM analogues and bile acid derivative are elevated in ASMD. ^c^I-cell disease (ML-II and -III) gives a false positive result (very different clinical features). ^d^ASMD can give a similar filipin pattern. ^e^Check allele segregation by parental study or other test

Once NPC is suspected clinically, diagnosis can be confirmed by the combination of biochemical and molecular genetic studies [35]. In recent years, several plasma metabolites (cholestane-3β, 5α, 6β-triol, lyso-sphingomyelin isoforms and bile acid metabolites) have emerged as sensitive and specific diagnostic biomarkers for NPC and their study, completed by genetic analyses, should now be considered as the first line laboratory testing [35, 36]. The filipin test, although still very useful, is no longer considered as the primary tool. Figure 1 describes a revised laboratory diagnostic algorithm for NPC.

***What investigations should be performed in individual suspected with NPC?***

### Biomarkers

***Statements #14:*** *Assessment of biomarkers should be considered as a first-line test to screen for NPC. Three classes of biochemical markers are either currently in use (oxysterols; lyso-SM-509 and lyso-sphingomyelin) or are in development (bile acid derivatives).They can be used alone or in combination to enhance sensitivity and specificity. The diagnosis, however, must in all cases be confirmed by mutation analysis and if necessary, filipin test.*

- *Strength of recommendation: 1*
- *Level of evidence: B*
- *Experts opinion: completely agree (75%), mostly agree (13%), partially agree (13%), mostly disagree (0%) and completely disagree (0%).*

#### *Oxysterols* (cholesterol oxidation products)

The oxysterols cholestane-3β, 5α, 6β-triol (C-triol) and 7-ketocholesterol (7-KC), are well established and accessible to clinicians (implemented in > 30 clinical laboratories worldwide). Both are sensitive markers of NP-C, though C-triol is more specific [37, 38].

Potential limitations:

- Elevated values have been reported in other metabolic disorders, particularly acid sphingomyelinase deficiency and lysosomal acid lipase deficiency, and to a lesser degree Cerebrotendinous xanthomatosis and Smith Lemli Opitz syndrome. These false positive results may reflect artefacts of the derivatization process used in the assay.
- Spurious values in the setting of neonatal cholestasis may result from assay interference. In this clinical setting, plasma bile acids are test of choice (see below).
- Overlap between upper quartile of NPC1 carriers and NPC1 patients must be considered when screening populations with low carrier frequency (e.g., general population).

#### Lyso-sphingolipids

The simultaneous mass spectrometric measurement in plasma of lyso-sphingomyelin (lyso-SM) (also known as sphingosyl-phosphorylcholine) [39] and of an analogue of unclear structure named lyso-sphingomyelin 509 (lyso-SM 509) appears as a very promising tool for initial screening of patients with either NPC or acid sphingomyelinase deficiency (ASMD) [39–44]. Striking elevations of Lyso-SM 509 have been reported in both NPC and ASMD, with high sensitivity for detecting both disorders, but poor distinction between them. For lyso-SM, a large increase only occurs in ASMD, with marginal or no elevation in NPC. Therefore, the combined assay of lyso-SM 509 and lyso-SM provides a good discrimination between NPC and ASMD. A high lysoSM-509/lysoSM ratio appears very specific of NPC. Further simultaneous measurement of other lysosphingolipids (e.g. glucosylsphingosine) in the same analysis can also discriminate other sphingolipidoses of clinical relevance – particularly Gaucher disease [42, 44].

Potential limitations:

- The structure of lyso-SM-509 is unknown, and exact concentrations cannot be measured
- Experience of clinical laboratories with Lyso-SM-509 and lyso-SM is still limited.
- Specificity of lyso-SM-509 among sphingolipidoses other than NPC and ASMD appears good [42–44] but will require further studies.
- It is still unclear whether this biomarker can discriminate between NPC1 carriers and NPC1 patients, which has implications for population screening.

#### Bile acids

Several unusual bile acid species in plasma and urine have been identified in NPC. The most important analytical species is 3β, 5α, 6β-trihydroxy-cholanoyl-glycine, detectable in plasma and dried blood spots [45, 46].

Potential benefits:

- It is highly sensitive and more specific than oxysterols (only elevated in acid sphingomyelinase deficiency and NPC).
- From available data, it provides complete discrimination between NPC1 carriers and NPC1 patients, suggesting it may be a biomarker of choice for population screening.
- It does not require derivatization and is less prone to interferences in the context of neonatal cholestasis, suggesting it may be the test of choice for diagnosing NPC disease in this clinical subset.
- This biomarker has greater stability than C-triol and can be shipped to diagnostic laboratories at ambient temperature.

Potential limitations:

- Test is new and performed at present in only a couple of research institutions.
- The biomarker has not been validated prospectively in clinical laboratories.

Good correlation has been observed between levels of the 3 biomarkers cholestane-triol, trihydroxycholanoyl-glycine and lyso-SM-509 in the same patient, but, from limited data, not necessarily with the level of lysosomal cholesterol storage observed in the filipin test.

### Molecular genetic studies:

***Statements #15:*** *Any individual in whom the diagnosis of NPC is considered based on their clinical manifestation and/or abnormal biomarker profile should undergo genetic testing for NPC genes to confirm the diagnosis. Referral to a clinical geneticist or genetic counsellor should be considered upon the diagnosis of NPC.*

- *Strength of recommendation: 1*
- *Level of evidence: A*
- *Experts opinion: completely agree (81%), mostly agree (19%), partially agree (0%), mostly disagree (0%) and completely disagree (0%).*

Mutation analysis of *NPC1* and *NPC2* genes is mandatory to confirm the diagnosis of NPC. In addition, it is the only reliable method to diagnose NPC carriers within the family and the highly preferred strategy for prenatal diagnosis. Furthermore, it may be useful to establish genotype-phenotype correlations. However, some genetic changes (e.g. deep intronic mutations, large deletions/ duplications) may not be identified by routine sequencing methods and will require complementary testing [47]. Nonetheless, a small proportion of mutated *NPC1* alleles have remained unidentified in proven patients. The segregation of the alleles should be confirmed in parents. The identification of two alleles with known disease-causing mutations in either *NPC1* or *NPC2* gene confirms the diagnosis of NPC. To date, about 700 *NPC1*variants have been reported, among which around 420 are considered as pathogenic, with only a limited number of common (p.I1061T, p.P1007A) or recurrent (often in certain populations) mutations. Thus, the interpretation of new missense and splicing mutations should be undertaken with caution and their pathogenic nature must be verified.

Although genotype/phenotype correlations are difficult to establish, some conclusions can be drawn from current evidence:

The presence in both alleles of a very severe mutation (frameshift, nonsense, large deletion) usually results in early-infantile neurological disease (with also a higher risk of severe – possibly fatal – neonatal systemic disease). From observations in homozygous patients, an increasing number of recurrent *NPC1* missense mutations (e.g. p.G1240R) can also be classified in this category.

The p.I1061T *NPC1* mutation [48, 49] has been reported in a large number of individuals; in homozygosity, it has mostly been associated with a juvenile neurological onset, less frequently with a late infantile neurological phenotype. In heterozygosity, depending on the second mutation, it is also often found in patients with an adolescent/adult onset form [13].

The p.P1007A *NPC1* mutation appears more commonly associated with a juvenile or adult onset form than with a late infantile one [22, 50]. Patients carrying this mutation (even on one allele) have been difficult to diagnose using the filipin test, as p.P1007A is the prototype of *NPC1* mutations associated with a less severe block in cholesterol egress from lysosomes, resulting in the so-called “variant” filipin phenotype [50].

Lately, an increasing number of recurrent *NPC1* missense mutations associated with a late-onset neurological form (e.g. p.R978C, p.G992R, p.D874V), have been reported even when found in compound heterozygosity with a severe or null allele.

Thus far, 26 pathogenic *NPC2* mutations have been described. Most are frameshift or nonsense as well as a large deletion variants leading to a severe clinical phenotype. Among the reported missense mutations, two variants (p.V39 M and p.P120S) have been associated with the juvenile or adult forms of the disease. More patients originating from North Africa, Italy and Turkey have been described with *NPC2* mutations.

Finally, studies of numerous multiplex families have indicated that mutations (either *NPC1* or *NPC2*) appear largely predictive of the neurological course, and not of the systemic disease.

### The Filipin test

***What is the diagnostic role of the filipin test in the era of new biomarkers?***

***Statements #16:*** *Filipin test is no longer considered a first line test for the diagnosis of NPC. It still remains an extremely useful diagnostic tool in uncertain cases in which biomarkers and/or molecular analysis present inconclusive results and to assess the pathogenicity of novel genetic variants.*

- *Strength of recommendation: 1*
- *Level of evidence: A*
- *Experts opinion: completely agree (81%), mostly agree (19%), partially agree (0%), mostly disagree (0%) and completely disagree (0%).*

Until recently, the demonstration of unesterified cholesterol accumulation within the lysosomes by filipin testing was considered to be the gold standard test for the diagnosis of NPC disease [11, 47, 51]. Since this assay needs to be performed on cultured fibroblasts obtained from skin biopsies, it is invasive and has a long turnaround time. Additionally, the assay is technically challenging, labour intensive and is performed only in specialized laboratories [51]. Due to these drawbacks, and in the light of the recent discovery of several sensitive and specific blood biomarkers, filipin staining is no longer considered as a first line test for the diagnosis of NPC [35]. However, it is very useful to assess the pathogenicity of novel genetic variants. It also remains a useful diagnostic tool in uncertain cases in which biomarkers and/or molecular analysis present inconclusive results. In particular, it is important to keep in mind that to date all biomarkers, except bile acids which have not yet been fully validated, do not completely differentiate between heterozygous and affected patients [39, 52, 53]. In these cases, if only one pathogenic mutation is found by molecular analysis of *NPC1* and *NPC2*, filipin testing should be performed. In some cases, distinction between a “variant” filipin profile and the slightly abnormal filipin profile observed in a number of NPC heterozygotes may however be difficult. Consequently, in a few patients, it may not be possible to definitively conclude in spite of comprehensive investigations.

### Brain imaging

***What is the role of brain imaging in the diagnosis and follow-up of NPC?***

***Statements #17:*** *Brain imaging changes in individuals with NPC are variable and non-specific, but the most commonly reported changes are reductions in volume of the cerebellum, hippocampus, and subcortical grey matter, in addition to subtle changes in most white matter regions.*

- *Strength of recommendation: 1*
- *Level of evidence: B*
- *Experts opinion: completely agree (47%), mostly agree (53%), partially agree (0%), mostly disagree (0%) and completely disagree (0%).*

Neuroimaging data gathered primarily from adolescent and adult individuals with NPC show a variable pattern, with some being normal, particularly early in the course of the illness, while most patients will demonstrate cerebellar volume changes, which correlate with measures of ataxia and ocular-motor function [54]. Reductions in the volume of the hippocampus, basal ganglia and thalamus are also associated with progressive illness [55]. White matter disease is often widespread, most detectable as changes on diffusion imaging [56] or visually as atrophy of the corpus callosum [57]. Increased pontine to mid-brain ratio, much like that seen in progressive supranuclear palsy, is seen, albeit to a lesser degree [58]. In some patients, brain atrophy may predominantly affect frontal and temporal regions [59]. However, these changes are often subtle and non-specific, and may be more useful as illness biomarker than as a diagnostic tool.

## Managment

NPC disease is not yet curable but is an eminently treatable condition. Optimal disease management employs a multi-disciplinary, multi-professional team based in a specialist centre, closely liaising with community care providers (Table 5). The mainstay of therapy is symptom management employing disease modifying agent(s) when available.

**Table 5:** Multidisciplinary assessments of patients with NPC

Discipline	Features of NPC for which this discipline may be of assistance	Reference
Primary care physician	Assist with general medical care; coordinate specialists; provide support for family	Expert opinion
Metabolic diseases specialist	Diagnosis of NPC and exclusion of other disorders in the differential diagnosis; ongoing patient assessment for disease progression and response to therapy	[47]
Neurologist	Cataplexy, movement disorders, dystonia, and seizures	[60]
Psychiatrist	Psychosis, behavioural disturbances, depression	[26]
Neuro-ophthalmologist	Diagnosis (vertical gaze palsy) and assess response to therapy (changes in saccadic eye movement velocity)	[61]
Anaesthesiologist	Assess for anaesthetic risk as needed	[76, 77]
Neuropsychologist	Assess for cognitive involvement at baseline and in response to therapy	[63]
Speech and language therapist	Assess for dysphagia and aspiration risk; speech therapy for children	Expert opinion
Occupational and physical therapists/Rehabilitation physician	Assess development and develop aids and home adjustments as needed for patients with communication and physical challenges	[64]
Orthopaedic surgeon	Assess the need for surgical correction of severe scoliosis, osteo-articular retractions, spasticity treatments and hip problems.	Expert opinion
Nutritionist/Gastroenterologists	Assess nutritional status in patients who may be losing weight due to dysphagia or side effects of therapy; Gastrostomy tube insertion when swallowing is unsafe.	[65]
Social worker	Support of patients and families living with disabilities who require enhanced resources in the community	Expert opinion
Genetic counsellor	Provide counselling for families as to recurrence risk and options for prenatal diagnosis if desired	[47]

***Statements #18:*** *Patients with NPC exhibit multisystem disease manifestations and benefit from multidisciplinary follow up from physicians and allied health care professionals with experience in this condition. Wherever possible, patients identified with NPC should be referred to a centre with expertise in the care of this condition.*

- *Strength of recommendation: 1*
- *Level of evidence: A*
- *Experts opinion: completely agree (100%), mostly agree (0%), partially agree (0%), mostly disagree (0%) and completely disagree (0%).*

### Symptomatic therapy

***What symptomatic therapy should be considered for a patient with NPC disease?***

The following functional assessments should take place at the time of diagnosis or symptom onset and at regular intervals thereafter for optimal symptom control and functional capacity (Table 6).

**Table 6:** Recommended assessments

Recommended assessment	Rationale	Frequency	References
Baseline history	Establish current level of disease severity and retrospectively estimate rate of progression	At diagnosis	[9, 62, 78]
Interval history	Establish rate of disease progression; monitor for compliance with and side effects from therapy; monitor for conditions which would prompt discontinuation of therapy	6 months	[62, 78]
Physical examination	Document growth parameters, assess for neurological features and organomegaly	At diagnosis then every 6–12 months	[61, 62]
NPC clinical severity score	Document key features of disease at diagnosis, progression and response to therapy	At diagnosis and then every 6 months	[22, 24, 32, 61]
Neuropsychiatric evaluation	Document and treat psychiatric manifestations and response to therapy	At diagnosis then every 6–12 months	[62, 79]
Developmental or cognitive assessment	Document baseline degree of cognitive impairment and monitor response to therapy	At diagnosis; every 6 months in children; every 12 months in adults	[9, 47, 61, 80]
Ophthalmology evaluation	Document saccadic eye movement velocity and presence of gaze palsy at baseline and document response to miglustat therapy in treated patients	At diagnosis; at 6 and 12 months; after starting treatment; frequency after 12 months can be determined by clinical response	[61]
Audiometry	Document presence of hearing loss	At diagnosis then every 12 months	[81]
Swallowing assessment	Clinical swallowing assessment in all patients; videofluoroscopic swallowing (VFS) assessment may be useful in some patients; Document presence of dysphagia and aspiration and response to therapy	At diagnosis and then every 6 months in children; in adults, frequency could be reduced to every 12 months if asymptomatic and disease is stable	[61, 82]
Neuroimaging	Magnetic resonance imaging or more detailed forms of neuroimaging including MR spectroscopy and diffusion tensor imaging	At baseline if available; Decisions about follow up neuroimaging will depend on local availability and need for general anaesthesia	[47, 83–85]

#### Growth and developmental delay

***Statements #19:*** *The growth of children with NPC (height, weight and head circumference) should be assessed at regular intervals as part of routine health assessments by their primary health care provider. In addition, their developmental progress should be monitored using age appropriate instruments.*

- *Strength of recommendation: 1*
- *Level of evidence: B*
- *Experts opinion: completely agree (80%), mostly agree (20%), partially agree (0%), mostly disagree (0%) and completely disagree (0%).*

#### Mobility

***Statements #20:*** *Mobility, balance, core stability, trunk control, spasticity, foot posture and strength should be assessed regularly by a suitably qualified physical therapist. Strategies to maintain optimal mobility and reduce falls such as providing an appropriate walking/mobility aids, ankle-foot orthotics and exercise programs should be sought proactively. A structured and personalized rehabilitation program may prolong mobility and transfer ability.*

- *Strength of recommendation: 1*
- *Level of evidence: B*
- *Experts opinion: completely agree (67%), mostly agree (33%), partially agree (0%), mostly disagree (0%) and completely disagree (0%).*

#### Swallowing and diet

***Statements #21:*** *NPC patients should undergo a comprehensive swallowing assessment by a speech and language therapist and nutritional review by a dietician. Instruction in dietary modification and compensatory postures may be beneficial for individuals with dysphagia. The family should be educated regarding the likely eventual need for assisted feeding, as part of an ongoing process.*

- *Strength of recommendation: 1*
- *Level of evidence: B*
- *Experts opinion: completely agree (86%), mostly agree (14%), partially agree (0%), mostly disagree (0%) and completely disagree (0%).*

#### Speech

***Statements #22:*** *NPC patients should undergo a comprehensive communication evaluation by a speech and language therapist and receive appropriate treatment.*

- *Strength of recommendation: 1*
- *Level of evidence: B*
- *Experts opinion: completely agree (87%), mostly agree (7%), partially agree (7%), mostly disagree (0%) and completely disagree (0%).*

#### Spasticity

***Statements #23:*** *Individuals with NPC may benefit from assessments for spasticity and incipient or established contracture. Spasticity and spasms should be treated at an early stage, initially by non-pharmacological means. If these are unsuccessful, pharmacological agents including Baclofen, Tizanidine, Benzodiazepines, Dantrolene sodium and botulinum toxin injections may be considered.*

- *Strength of recommendation: 1*
- *Level of evidence: B*
- *Experts opinion: completely agree (93%), mostly agree (7%), partially agree (0%), mostly disagree (0%) and completely disagree (0%).*

#### Bowel dysfunction

***Statements #24:*** Co*nsider modifying diet and lifestyle to optimize stool consistency and avoid faecal impaction and incontinence. If required, consider appropriate laxatives to optimize gut transit and stool consistency.*

- *Strength of recommendation: 1*
- *Level of evidence: B*
- *Experts opinion: completely agree (80%), mostly agree (20%), partially agree (0%), mostly disagree (0%) and completely disagree (0%).*

#### Bladder dysfunction

***Statements #25:*** *Individuals with NPC should have their history reviewed for symptoms suggestive of neurogenic bladder (recurrent urinary tract infection, nocturia, incomplete evacuation, dribbling) and be referred for urologic evaluation if symptoms are present.*

- *Strength of recommendation: 1*
- *Level of evidence: B*
- *Experts opinion: completely agree (79%), mostly agree (21%), partially agree (0%), mostly disagree (0%) and completely disagree (0%).*

#### Cataplexy and seizures

***Statements #26:*** *Cataplexy and seizures are common manifestations of NPC and their early recognition is important and should be managed promptly as per local/national management guidelines. Protriptyline, other tricyclic agents or modafanil have been efficacious for cataplexy. Epilepsy should be treated by a neurologist aware of the disease (possibility of aggravation with antiepileptic drugs like carbamazepine and vigabatrin should be considered).*

- *Strength of recommendation: 1*
- *Level of evidence: B*
- *Experts opinion: completely agree (62%), mostly agree (38%), partially agree (0%), mostly disagree (0%) and completely disagree (0%).*

#### Cognitive decline

***Statements #27:*** *Individuals with NPC benefit from regular evaluation of their cognitive function and consideration should be given to changes in their cognitive ability that may impact on independence/school/work and daily living activities. Testing should be age and functionally appropriate, using standardised assessment tools. Strategies to ensure the safety of the patient’s environment and the availability of support mechanisms are essential to improve the quality of life of the patient*.

- *Strength of recommendation: 1*
- *Level of evidence: B*
- *Experts opinion: completely agree (80%), mostly agree (13%), partially agree (7%), mostly disagree (0%) and completely disagree (0%).*

#### Mental wellbeing

***Statements #28:*** *Clinicians, caregivers and individuals with NPC should be aware that there is an increased prevalence of behavioural problems and other psychiatric disorders such as anxiety, depression or psychosis in NPC. There should be a low threshold for referral to a clinical psychology/psychiatric team as appropriate, and for the use of both non-pharmacological and/or pharmacological treatments.*

- *Strength of recommendation: 1*
- *Level of evidence: B*
- *Experts opinion: completely agree (93%), mostly agree (7%), partially agree (0%), mostly disagree (0%) and completely disagree (0%).*

#### Hypersalivation / drooling

***Statements #29:*** *Individuals with NPC are at increased risk of hypersalivation/drooling and should be treated with established interventions including postural drainage +/−pharmacological agents such as Hyoscine hydrobromide transdermal patches; Glycopyrronium orally, subcutaneously or* via *a gastrostomy and small doses of orally administered atropine, or parotid/submandibular glandular injections of botulinum toxin.*

- *Strength of recommendation: 1*
- *Level of evidence: B*
- *Experts opinion: completely agree (73%), mostly agree (27%), partially agree (0%), mostly disagree (0%) and completely disagree (0%).*

#### Hearing

***Statements #30:*** *Individuals with NPC should undergo a comprehensive hearing assessment at the time of diagnosis and thereafter annually. When appropriate, patients should be offered hearing devices to improve general communication.*

- *Strength of recommendation: 1*
- *Level of evidence: B*
- *Experts opinion: completely agree (67%), mostly agree (20%), partially agree (13%), mostly disagree (0%) and completely disagree (0%).*

### Disease modifying therapy

#### Miglustat

Miglustat, a substrate reduction therapy, is the only licensed disease modifying medicine in the European Union for the treatment of neurological manifestations of patients with NPC disease. In some patients, miglustat has been shown to halt or attenuate disease progression [60, 61].

##### Miglustat start criteria

**Statement # 31:** *All patients with a confirmed diagnosis of NPC should be considered for miglustat therapy*

- *Strength of recommendation: 2*
- *Level of evidence: C*
- *Experts opinion: completely agree (13%), mostly agree (38%), partially agree (13%), mostly disagree (25%) and completely disagree (13%).*

To understand the effects of disease modifying therapy in NPC, information about the natural history of disease progression is required. In one natural history of NPC, a cohort of 57 NPC patients was analysed, with 85.7% who were followed up for more than 1 year showed neurological disease progression [62]. In this cohort were children < 6 years who had normal evaluation suggesting they might have had a late onset phenotype. The rate of progression was 0.12 points per year (CI 0.09 to 0.15) using a composite NPC scoring system where the maximum score of 4 indicates severe disease. The rates of progression correlated with age at diagnosis, the younger patients showing the greatest progression of disease.

A phase I/II study of miglustat was performed in 29 patients ≥12 years of age with proven NPC [61] Patients were randomised 2:1 to receive miglustat 200 mg tds or standard care for 1 year, with the option for adult patients to enrol into a further 1 year extension study to receive active drug. A further subgroup of 12 patients ≤12 years received miglustat at a dose based on body surface area. The study included male and female patients with NPC confirmed by cholesterol esterification and abnormal filipin staining, able to safely ingest a capsule, with normal renal function and not suffering from clinically significant diarrhoea. Patients with other medical conditions or were on concomitant medications that would render them unsuitable for the study were excluded. Patients were assessed for the primary end point – change in horizontal saccadic eye movements (HSEM) at baseline and at 12 months. At each assessment of eye movement velocity was tested twice during a 24 h period. Swallowing ability was assessed at screening, 6 months and 12 months. Neurological assessments and quality of life assessments were performed at screening, 3, 6, 9 and 12 months.

Treatment with Miglustat resulted in improvements in the primary end point (HSEM) compared with standard care. At 12 months, HSEM velocity had improved in patients treated with miglustat versus those receiving standard care; results were significant when patients taking benzodiazepines were excluded (*p* = 0.028). Children showed an improvement in HSEM velocity of similar magnitude at 12 months. Improvement in swallowing capacity, stable auditory acuity, and a slower deterioration in ambulatory index were also seen in treated patients older than 12 years. Safety assessments were performed at screening every 3 months and at post screening follow up. Adverse events (AEs) were recorded at each post-screening visit. The most frequently occurring AEs were diarrhoea (85%), flatulence (70%), and weight loss (65%). Discontinuation was reported in one paediatric patient due to memory impairment and in one adult patient due to confusional state and in one other adult patient due to diarrhoea. No deaths were reported. The study concluded that Miglustat was safe and improved or stabilised several clinically relevant markers of NPC [61]. This is the first agent studied in NPC for which there is both animal and clinical data supporting a disease modifying benefit.

Longer term (24 month) data of patients in the above study, as part of an open label extension has been reported [63]. 19/29 patients from the pivotal study completed the two-year study of whom 15/19 completed 24 months of miglustat therapy. The 24-month data did not meet the primary end point of improvement in HSEM velocity. Small patient numbers produced wide confidence intervals making the data unreliable; however a modest deterioration in HSEM velocity was noted. Overall there was stabilisation of neurological symptoms (cognition, ambulation and swallow) in 68% of this group with a trend towards improvement when compared to the natural history data.

***Statement #32:*** *NPC patients who are pre-symptomatic or have only spleen/liver enlargement should not be offered miglustat.*

- *Strength of recommendation 2*
- *Level of evidence C*
- *Experts opinion: completely agree (27%), mostly agree (40%), partially agree (27%), mostly disagree (7%) and completely disagree (0%).*

All pre-symptomatic subjects should undergo regular evaluation by a neurologist and/or metabolic physician, so that treatment can be considered at an early onset of neurological manifestation.

***Statement #33:*** *Miglustat should not be started in NPC patients with advanced neurological disease/dementia.*

- *Strength of recommendation 2*
- *Level of evidence C*
- *Experts opinion: completely agree (47%), mostly agree (33%), partially agree (20%), mostly disagree (0%) and completely disagree (0%).*

Currently there are limited data on patients with NPC with advanced neurological disease being commenced on miglustat. Based on the French experience of miglustat treatment in 20 children the NPC disability scores improved or stabilized in 75% of the patients with late-infantile onset disease (onset of symptoms < 5 years of age) but no patients with the early infantile onset form (onset of symptoms < 1 year of age) had a good neurological outcome [64]. Only one patient out of 9 children treated before 4 years of age demonstrated stabilisation. More data are needed to determine of the efficacy of miglustat in patients below the age of 4 years.

Miglustat therapy is NOT appropriate for patients who have profound neurological disease, which, in the opinion of the attending physician, would make it difficult to assess for any improvements with therapy. Such symptoms may include but are not limited to:

- Profound dementia resulting in the need for 24 h care
- Inability to ambulate without a wheelchair
- Complete lack of verbal communication
- Swallowing difficulties profound enough to require tube feeding through a per-cutaneous gastrostomy

***Statement #34:*** *Miglustat should not be started in NPC patients with another life threatening illness with estimated life span less than 1 year.*

- *Strength of recommendation 2*
- *Level of evidence C*
- *Experts opinion: completely agree (73%), mostly agree (20%), partially agree (7%), mostly disagree (0%) and completely disagree (0%).*

Whilst there is no evidence in the literature to assess this, most guidelines for other new therapies such as enzyme replacement therapies, and some national guidelines specify the above as an exclusion criteria [65, 66].

#### Experimental therapies

Clinical trials testing the safety and efficacy of intrathecal [67] or intravenous preparations of 2-hydroxypropyl- β-cyclodextrin and oral Arimoclomol are ongoing. Although the disease is a disorder of cholesterol trafficking, cholesterol-lowering drugs have not been shown to be effective at altering the course of the disease [68, 69]. In addition, a number of other therapeutic modalities in animal and early phase human studies are underway.

### Follow up, transition, advanced care planning and genetic issues

#### Follow up

***Statements #35:*** *NPC is a progressive condition and patients require regular follow up. Treatment goals should be established at diagnosis and reviewed regularly, aimed at improving or maintaining the physical and psychosocial wellbeing of individuals with NPC and their families.*

- *Strength of recommendation: 1*
- *Level of evidence: B*
- *Experts opinion: completely agree (88%), mostly agree (6%), partially agree (6%), mostly disagree (0%) and completely disagree (0%).*

#### Transition

***Statements #36:*** *Most children with late-infantile and juvenile onset NPC are expected to reach adulthood with complex medical and psychosocial needs. The process of transition from paediatric to adult services should begin early and must include appropriate services in the community to provide a seamless transition from childhood to adult life. Individuals with NPC may benefit from a detailed assessment identifying barriers to independence.*

- *Strength of recommendation: 1*
- *Level of evidence: B*
- Experts opinion: completely agree (88%), mostly agree (13%), partially agree (0%), mostly disagree (0%) and completely disagree (0%).

#### Advance care planning

***Statements #37*** *: Specialist centre care providers, family physician/paediatrician and local palliative care services should develop close working links to support individuals and families with NPC through the lifespan, including: a) advance care planning with regular updating. b) proper flow of communication and information for patients and their families, c) a designated point of contact for each stage in their care pathway. An individual identified as being near the end- of-life may benefit from ongoing access to palliative care services including for symptom control, respite, psychological and spiritual support.*

- *Strength of recommendation: 1*
- *Level of evidence: B*
- *Experts opinion: completely agree (94%), mostly agree (6%), partially agree (0%), mostly disagree (0%) and completely disagree (0%).*

#### Genetic issues

***Statements #38*** *: Requests for NPC pre-symptomatic genetic testing are best managed on a case-by-case basis. Pre-symptomatic testing in minors is not permitted in some jurisdictions, and in any case, the risks and benefits from the perspectives of both the child and parents should be carefully discussed in the context of formal counselling from a suitably qualified individual. All patients identified pre-symptomatically should be referred to specialist centres for surveillance and early detection of neurological manifestations.*

- *Strength of recommendation: 2*
- *Level of evidence: B*
- *Experts opinion: completely agree (81%), mostly agree (6%), partially agree (13%), mostly disagree (0%) and completely disagree (0%).*

***Statements #39:*** *Prenatal testing for NPC should be offered to all at risk couples and requires careful counselling by clinical geneticists and NPC specialists. Molecular genetic analysis of chorionic villus samples is the strategy of choice, based on mutations identified in the family.*

- *Strength of recommendation: 1*
- *Level of evidence: B*
- *Experts opinion: completely agree (75%), mostly agree (13%), partially agree (6%), mostly disagree (6%) and completely disagree (0%).*

## Conclusion

These guidelines are the result of an international collaboration of experts in the care of NPC and the evidence gathered to write these guidelines is the best evidence available to the experts. These guidelines address the management of children and adults affected by NPC and are intended to facilitate optimal care to all NPC patients regardless of their demography and access to health care. In addition, it defines standard of care against which practice can be audited and best practice can be spread. The Guidelines Working Group commits itself to revise this work in 5 years’ time to reflect new data pertaining to future research findings and new therapies.

## Abbreviations

7-KC: 7-ketocholesterol
AE: Adverse Event
AGREE II: Appraisal of Guidelines for Research & Evaluation
ASMD: Acid Sphingomyelinase Deficiency
CHAFEA: Consumers, Health, Agriculture and Food Executive Agency
C-triol: Cholestane-3β, 5α, 6β-triol
DG-SANGO: EU Directorate General for Health and Consumers
GDG: Guidelines Development Group
GRADE: Grading of Recommendations, Assessment, Development and Evaluations
HSEM: Horizontal Saccadic Eye Movements
INPDA: International Niemann-Pick Disease Alliance
INPDR: International Niemann-Pick Disease Registry
lyso-SM 509: Lyso-sphingomyelin 509
MetabERN: European Metabolic Reference network
MRI: Magnetic Resonance Imaging
NPC: Niemann-Pick Type C
RCT: Randomized Controlled Trials
VSGP: Vertical supranuclear gaze palsy
